# HIV Infected T Cells Can Proliferate *in vivo* Without Inducing Expression of the Integrated Provirus

**DOI:** 10.3389/fmicb.2019.02204

**Published:** 2019-10-01

**Authors:** Andrew Musick, Jonathan Spindler, Eli Boritz, Liliana Pérez, Daniel Crespo-Vélez, Sean C. Patro, Michele D. Sobolewski, Michael J. Bale, Carolyn Reid, Brandon F. Keele, Adam Capoferri, Wei Shao, Ann Wiegand, Francesco R. Simonetti, John W. Mellors, Stephen H. Hughes, John M. Coffin, Frank Maldarelli, Mary F. Kearney

**Affiliations:** ^1^Human Immunodeficiency Virus (HIV) Dynamics and Replication Program, Center for Cancer Research (CCR), National Cancer Institute (NCI)-Frederick, Frederick, MD, United States; ^2^Virus Persistence and Dynamics Section, Vaccine Research Center, National Institute for Allergies and Infectious Diseases (NIAID), Bethesda, MD, United States; ^3^Department of Medicine, University of Pittsburgh, Pittsburgh, PA, United States; ^4^Frederick National Laboratory for Cancer Research, Aquired Immunodeficiency Syndrome (AIDS) and Cancer Virus Program, Leidos Biomedical Research Inc., Frederick, MD, United States; ^5^Frederick National Laboratory for Cancer Research, Advanced Biomedical Computing Center, Leidos Biomedical Research, Inc., Frederick, MD, United States; ^6^Department of Molecular Biology and Microbiology, Tufts University, Boston, MA, United States

**Keywords:** HIV reservoir, latent, latently-infected cells, cell-associated HIV RNA, expanded clones, proviral expression, CARD-SGS, SGA

## Abstract

**Background:**

HIV-1 proviruses can persist during ART in clonally-expanded populations of CD4+ T cells. To date, few examples of an expanded clones containing replication-competent proviruses exist, although it is suspected to be common. One such clone, denoted AMBI-1 (Maldarelli et al., 2014), was also a source of persistent viremia on ART, begging the question of how the AMBI-1 clone can survive despite infection with a replication-competent, actively-expressing provirus. We hypothesized that only a small fraction of cells within the AMBI-1 clone are activated to produce virus particles during cell division while the majority remain latent despite division, ensuring their survival. To address this question, we determined the fraction of HIV-1 proviruses within the AMBI-1 clone that expresses unspliced cell-associated RNA during ART and compared this fraction to 33 other infected T cell clones within the same individual.

**Results:**

In total, 34 different clones carrying either intact or defective proviruses in “Patient 1” from Maldarelli et al. (2014) were assessed. We found that 2.3% of cells within the AMBI-1 clone contained unspliced HIV-1 RNA. Highest levels of HIV-1 RNA were found in the effector memory (EM) T cell subset. The fraction of cells within clones that contained HIV-1 RNA was not different in clones with intact (median 2.3%) versus defective (median 3.5%) proviruses (*p* = 0.2). However, higher fractions and levels of RNA were found in cells with proviruses containing multiple drug resistance mutations, including those contributing to rebound viremia.

**Conclusion:**

These findings show that the vast majority of HIV-1 proviruses within expanded T cell clones, including intact proviruses, may be transcriptionally silent at any given time, implying that infected T cells may be able to be activated to proliferate without inducing the expression of the integrated provirus or, alternatelively, may be able to proliferate without cellular activation. The results of this study suggest that the long, presumed correlation between the level of cellular and proviral activation may not be accurate and, therefore, requires further investigation.

## Introduction

HIV-1 replication is likely efficiently halted with antiretroviral therapy (ART), which prevents disease progression, but ART does not cure the infection (Persaud et al., 2007; Josefsson et al., 2013; Kearney et al., 2014; Maldarelli et al., 2014; Wagner et al., 2014; Palma et al., 2016; Vancoillie et al., 2017; Mok et al., 2018). Proliferation of cells, most likely infected prior to ART initiation, that carry replication-competent proviruses (those that are fully intact and capable of producing infectious virus particles) is an important mechanism for maintaining the HIV-1 reservoir (Lorenzi et al., 2016; Simonetti et al., 2016). It has been proposed that these cells are latently-infected and consequently transcriptionally silent, allowing them to evade virus-induced cytopathogenicity and immune-mediated clearance (Chun et al., 1995; Siliciano, 1999; Simonetti et al., 2016). By this model, persistent and rebound viremia originates from the occasional activation of a small fraction of the pool of latently-infected cells. It has been suggested that latently-infected cells may be killed upon such activation (Spina et al., 2013; Pollack et al., 2017), although some studies have challenged this idea (Deng et al., 2015; Bui et al., 2017).

Little is known about the fraction of infected cells persisting on ART that are latently-infected versus transcriptionally active or about the factors that influence proviral transcription. However, recent studies demonstrated that a high fraction of infected cells can initiate proviral transcription on ART but that a block to transcriptional elongation exists (Yukl et al., 2018) and that different types of proviral defects can affect their transcription (Pinzone et al., 2019). We recently developed the cell-associated RNA and DNA single-genome sequencing assay (CARD-SGS) (Wiegand et al., 2017) that can be used to quantify the proportion of infected cells producing HIV-1 RNA and to estimate the levels of expression in such cells. Preliminary studies suggested that during *in vivo* infection, greater than 80% of HIV-1 infected cells have proviruses that are transcriptionally-silent after long-term ART and that cells harboring transcriptionally-active proviruses contain only low levels of unspliced cell-associated HIV-1 RNA (median 1 ca-HIV RNA/cell) (Wiegand et al., 2017). However, the fractions of transcriptionally-silent proviruses versus transcriptionally-active proviruses remained unknown within populations of clonally-expanded infected cells, each of which contains the identical provirus at the identical site of integration, including those that carry intact proviruses (Simonetti et al., 2016; Einkauf et al., 2019). Furthermore, it is also not known which CD4+ T cell subsets expand and support the expression of HIV-1 proviruses that persist on ART, although effector memory (EM) cells have been suggested (Hiener et al., 2017; Pardons et al., 2019).

To date, few examples of an expanded clones containing replication-competent proviruses exist. However, one such clone, denoted AMBI-1 (Maldarelli et al., 2014), was shown, not only to contain an intact provirus, but to be the primary source of persistent viremia on ART in this individual, begging the question of how the AMBI-1 clone can survive despite infection with a replication-competent, actively-expressing provirus. We hypothesize that the AMBI-1 clone is able to persist because only a small fraction of cells within the clone are activated to produce virus particles during cell division while the majority remain latent despite division, ensuring their survival. Such a finding might imply that infected T cells can be activated to proliferate without inducing the expression of the integrated provirus or, alternatelively, may be able to proliferate without cellular activation.

To address this question, we investigated peripheral blood mononuclear cells (PBMC) from a patient who presented with low level detectable viremia after prolonged ART. Previous analyses revealed that the on ART viremia in this individual originated from two sources: (1) viral replication of drug-resistant variants and (2) virus expression from a highly expanded T cell clone harboring a replication-competent, wild-type HIV-1 provirus denoted AMBI-1 (Maldarelli et al., 2014; Simonetti et al., 2016). Cells containing AMBI-1 comprised the largest infected cell clone in this individual (approximately 10^7^ cells) and was the sole source of wild-type persistent viremia during ART (Simonetti et al., 2016). We investigated samples from this patient to measure levels of HIV production both from cells infected via possible ongoing replication (drug resistant virus) and from long-lived reservoirs (wild-type virus). We identified a total of 34 different wild-type infected cell clones and possible clones (proviruses that are identical in P6-PR-RT), and used CARD-SGS (Wiegand et al., 2017) to determine the fraction of PBMC within each clone, including the AMBI-1 clone, that had detectable amounts of ca-HIV RNA. A methods paper on CARD-SGS was previously published and was shown to detect a single unspliced RNA molecule in a single cell (Wiegand et al., 2017). We also examined if the nature of the provirus (intact or defective) was associated with the fraction of infected PBMC that contained ca-HIV RNA and we quantified the levels of ca-HIV RNA in single infected cells in each of the 34 different infected cell clones and in cells infected with drug resistant variants. We determined that a relatively small proportion of PBMC produce ca-HIV RNA, and within a clone of identical cells, on average, less than 10% are producing ca-HIV RNA at any given time. Similar fractions and levels of expression were observed from clones harboring replication-competent proviruses or defective proviruses. However, higher fractions and levels of expression were observed in cells infected from probable ongoing viral replication of drug resistant variants.

After determining the fraction and levels of unspliced HIV-1 RNA in the 34 different infected cell clones and in the populations of PBMC infected with drug resistant variants, we sought to determine the specific CD4+ T cell subsets that harbored each of these clonal populations, especially those with intact proviruses, and to identify the cell subsets that supported their expression. We found possible clonal populations to be present in central/transitional memory (CTM) and EM T cell subsets, however, a higher fraction was found in EM cells, including in the clones harboring replication-competent proviruses.

## Results and Discussion

### Participant Description, Phylogenetic Analysis of Plasma Viremia, and Viral Outgrowth Assays

**FIGURE 1:**
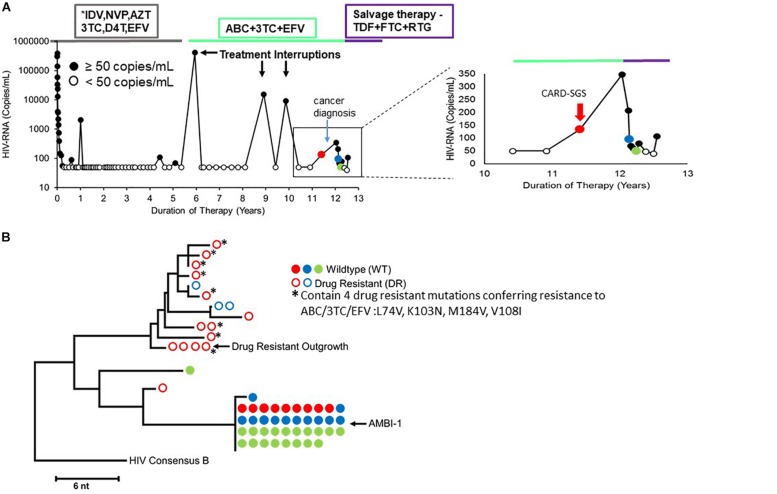
**(A)** Longitudinal viral load plot of Patient 1 in Maldarelli et al. (2014) and Simonetti et al. (2016) from the time of ART initiation to his death from cancer. Viral loads below limit of detection (<50 copies per mL) are indicated with open black circles. The red arrow in the right panel indicates the timepoint that was analyzed by CARD-SGS (viral load of 134 copies/ml). **(B)** NJ tree showing plasma RNA sequences at three marked timepoints on the viral load plot in A. Solid circles indicate sequences with no drug resistance mutations and open circles indicate drug-resistant variants. The ART regimens employed are shown in the boxes at the top. ^∗^Indicated that the drug regimen was simplifed for the figure.

The patient (Maldarelli et al., 2014; Simonetti et al., 2016) was an African-American male who was diagnosed with advanced HIV-1 infection (Deng et al., 2015) CD4+ T cells/μl) in May of 2000. He was treated with ART for 13 years; however, unplanned treatment interruptions did occur (Figure 1A; Simonetti et al., 2016). While on ART, the HIV plasma RNA levels declined to below the limit of detection (less than 50 HIV-RNA copies/ml) and CD4+ T cell count partially recovered. However, low-level rebound viremia occurred after about 11 years on ART (Figure 1) that was coincident with the development of a squamous cell carcinoma.

To investigate HIV populations in this low-level viremia, a neighbor joining (NJ) phylogenetic distance tree was constructed using data obtained by P6-PR-RT SGS from plasma samples taken at three timepoints immediately before and after the ART regimen change (Figure 1B). The presence of drug-resistant (DR) virus prompted a change in ART regimen that re-suppressed drug-resistant viremia, while the identical wild type (WT) sequences persisted (Figure 1). These data indicated that, as expected, the ART-resistant virus was the product of ongoing replication and likely recombination (Lu et al., 2018), while the WT virus population was produced from a reservoir of long-lived cells. Sampling allowed the investigation of HIV production from clonally-infected, long-lived cells (WT virus) as well as from ongoing replication (DR virus). The DR population was highly diverse indicating recent viral replication, whereas the WT population consisted almost entirely of identical sequences that were previously shown to arise from a highly-expanded clone of CD4+ T cells, called AMBI-1 (Maldarelli et al., 2014; Simonetti et al., 2016). Of note, previous studies demonstrated a lack of evidence for viral replication on ART in this patient prior to treatment interruptions (Kearney et al., 2014) (PID11 is Patient 1 in Kearney et al., 2014) and demonstrated the emergence of drug resistance mutations during and after treatment interruptions (Simonetti et al., 2016). Supplementary Figure S2 combines the drug resistance sequences from Simonetti et al. (2016) with those obtained here to show the detection of drug resistance mutations as early as 5 years prior to virologic failure.

An endpoint-dilution, single-stimulation, viral outgrowth assay (VOA) performed at the timepoint marked in red revealed four different replication-competent proviruses: WT AMBI-1, WT Outgrowth-1, WT Outgrowth-2, and DR Outgrowth-1 (Simonetti et al., 2016). Sequences matching two of these outgrowth viruses, AMBI-1 and DR Outgrowth, were also previously detected in plasma (Figure 1B). These data suggest that both WT and DR virus are capable of *ex vivo* outgrowth and, likely, *in vivo* rebound if ART is interrupted.

### HIV Proviral Sequences Reveal Possible Clones in PBMC

**FIGURE 2:**
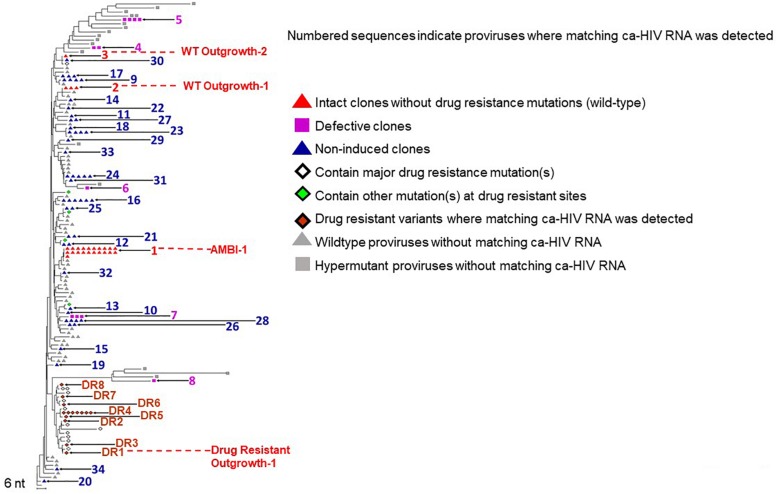
NJ distance tree of PBMC viral DNA sequences from the timepoint indicated in red in Figure 1. The tree contains possible clones with matching ca-HIV RNA (indicated with numbers) including the replication-competent clone AMBI-1, and the possible clones Outgrowth-1 and Outgrowth-2 in red, defective clones in purple, non-induced possible clones in blue, and DR proviruses with matching ca-HIV RNA in orange. Proviruses where no ca-HIV RNA was detected are shown in gray. A total of 183 proviral sequences were analyzed.

To characterize HIV populations in cells, CARD-SGS was performed on PBMC collected from the first timepoint (Figure 1A, red) shortly after breakthrough viremia on ART, at which time the plasma contained a mixture of about 50% DR and 50% WT (mostly AMBI-1) (Figure 1B). Leukapheresis could not be performed after the ART regimen shift due to the cancer diagnosis. CARD-SGS was previously shown to be able to detect a single intact, unspliced RNA molecule in a single cell by diluting the cells to an endpoint for those with HIV RNA, extracting the RNA and synthesizing cDNA in a single tube, and performing nested PCR and sequencing (Wiegand et al., 2017). Subgenomic, rather than near full-length, sequencing was used to ensure that every RNA molecule was detected as described in Wiegand et al. (2017). RNA may be sheered upon free-thawing of PBMC and, therefore, it is likely that near full-length sequencing methods could under estimate the number of unspliced HIV RNA molecules in a cell. Prior to investigating cell-associated HIV RNA, we obtained 183 independent P6-PR-RT proviral sequences from this timepoint and constructed a NJ distance tree (Figure 2). Because the total HIV diversity in this individual was very high (2.9% average pairwise distance) as previously reported (Maldarelli et al., 2014), the probability of obtaining two identical P6-PR-RT sequences by chance (not due to clonal expansion or a genetic bottleneck, such as the selection of drug resistance mutations) in our dataset was <10^–6^ (calculated using an in-house program that considers both the average pairwise distance, the length, and the distribution of the sequences^1^). We defined identical WT proviral sequences (or matching RNA sequences in different aliquots of cells) as “possible clones” of HIV-1 infected cells. Possible clones were divided into three categories: those carrying replication-competent proviruses determined by VOA (intact clones), those containing obviously defective proviruses (defective clones), and those having proviruses with no obvious defects in the region sequenced but were not recovered from the VOA (non-induced possible clones) (Simonetti et al., 2016). Of note, non-induced does not mean non-inducible since only a single round of VOA was performed. However, considering that >90% of proviruses persisting on ART are defective (Ho et al., 2013), the clones that were not induced in VOA are, statistically, more likely to be defective proviruses than to be intact.

We found ca-HIV RNA matching 34 of the clonal populations (Figure 2, numbered). For all clones, in all categories, a fraction of cells contained unspliced HIV-1 transcripts *in vivo*, including the WT replication-competent clones AMBI-1, Outgrowth-1, and Outgrowth-2 (red triangles). Consistent with results from the integration sites assay (ISA) (Maldarelli et al., 2014), the AMBI-1 clone was the largest detected by CARD-SGS. Five clones with matching ca-HIV RNA carried defective proviruses with stop codons (purple squares), indicating that defective proviruses can express ca-HIV RNA *in vivo*, as previously reported (Kearney et al., 2015a, b; Imamichi et al., 2016; Wiegand et al., 2017; Pinzone et al., 2019). Identical sequences were detected from 26 other distinct proviruses with matching ca-HIV RNA (blue triangles). These 26 possible clones did not have obvious defects (i.e., stop codons or large deletions) in the region sequenced but failed to produce detectable levels of infectious virus in a VOA despite the detection of ca-HIV RNA *in vivo*, suggesting defects elsewhere in the provirus or that their frequency was below the sampling capacity of a single round of VOA. Seventy-seven proviruses for which no matching ca-HIV RNA was detected, are shown with gray triangles for WT and squares for hypermutants. The fraction of RNA containing cells in these possible clones may have been below our level of detection or the proviral DNA may be integrated into a region that is less permissive to reactivation. As will be described in more detail below, we detected ca-HIV RNA matching each of the 3 intact proviruses, indicating that the reservoir for replication-competent HIV-1 contains cells with transcriptionally-active proviruses *in vivo* as well as with silent proviruses.

As expected, DR proviruses were also detected by this analysis. DR sequences constituted only 16% of the proviral population (Figure 2) but represented nearly half of the rebounding plasma virus (Figure 1B), indicating that only a small fraction of the total pool of infected cells contributed to half of viremia. The eight DR variants with matching ca-HIV RNA are indicated with closed orange diamonds. Positive VOA was observed for one of these proviruses, labeled “DR Outgrowth-1” (Figure 2). DR proviruses for which no ca-HIV RNA was detected are indicated with open diamonds (known DR mutations) or green diamonds (other mutations at DR sites). As in WT proviruses, we observed DR proviruses that were transcriptionally silent, transcriptionally active but not induced, and transcriptionally active and capable of *ex vivo* outgrowth and *in vivo* rebound.

### Possible Clones in CD4+ T Cell Subsets

**FIGURE 3:**
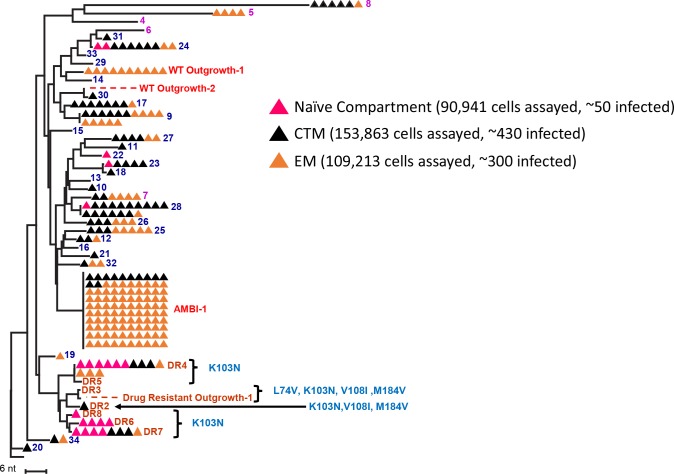
NJ distance tree of proviral sequences from CD4+ T cell memory subsets from the first timepoint (red) in Figure 1. PBMC were sorted into naïve (pink), central-transitional memory (black), and effector memory (orange) CD4+ T-cell subsets. Possible clones from which ca-HIV RNA was recovered are numbered, including VOA outgrowth viruses. DR mutations are indicated.

To determine the prevalence of the 34 possible clones in each subset, PBMC from the same timepoint (Figure 1) were sorted by markers defining them as naïve (or stem cells-like memory cells), CTM, and EM CD4+ T cells (Figure 3 and Supplementary Figure S1). Previous reports have claimed infection and clonal expansion in naïve cells (Venanzi Rullo et al., 2019; Zerbato et al., 2019), however, our sorting protocol did not distinguish stem cell-like memory cells from naïve so we cannot claim a pure naïve population. Twenty-seven of the 34 clones were detected in 90,000–150,000 cells assayed for each subset. The 27 clones identified were about equally represented in CTM (21/27) and EM (16/27) (*p* = 0.3, Fisher exact), but were less prevalent in possible naïve cells (4/27) (p_*Na*__ï__*ve,CTM*_ = 0.0001, p_*Na*__ï__*ve,EM*_ = 0.004, Fisher exact). Although the fraction of CTM and EM cells harboring unique DR variants was not different (*p* = 0.5, Fisher exact), a higher fraction of the infected naïve cells harbored DR variants than the CTM or EM subsets (naïve and CTM *p* = 1.2^∗^10^–4^; naïve and EM *p* = 7.8^∗^10^–5^, Fisher exact). Because naïve cells have higher surface expression of CXCR4 (Riley et al., 1998; Groot et al., 2006), it is possible that the DR mutations arose on a CXCR4-using backbone, explaining their higher prevalence in what could be naïve cells. *Env* sequencing and geno2pheno analyses (coreceptor.geno2pheno.org) confirmed that CXCR4-tropic envelopes were prevalent in the proviral population (sequences available in Genbank accession numbers MK148701-MK152485), however, due to the very low plamsa RNA levels, we could not link the drug resistance mutations in the plasma to their env tropism.

Importantly, the replication-competent provirus, AMBI-1, was found in both CTM and EM. Twelve of 100 AMBI proviruses sequenced in the cell subsets were in CTM and the other 88 in EM (Figure 3). Geno2pheno results on AMBI-1 *env* suggested CCR5-usage, possibly explaining its lack of detection in the naïve cell population. Ten Outgrowth-1 proviruses were identified in the subsets and all were in EM. These results suggest that, although clones may be equally prevalent in CTM and EM cells, the replication-competent proviruses may be more readily detected in the EM, consistent with previous findings by Hiener et al. (2017).

### Fraction and Levels of Cell-Associated HIV-1 RNA in Possible Cell Clones

**FIGURE 4:**
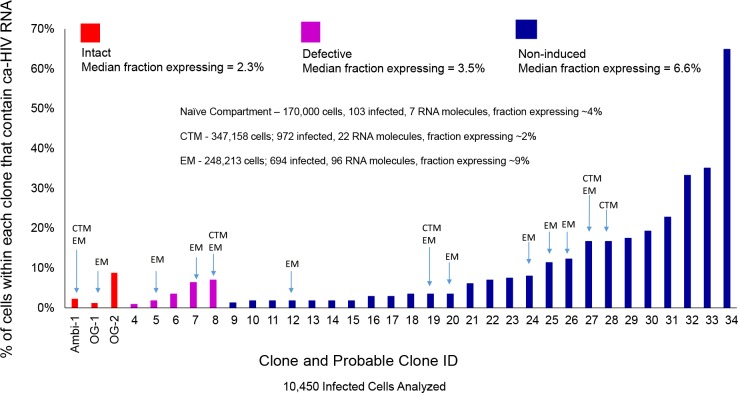
Plot of the fraction of cells that contained ca-HIV RNA for each of the 34 different clones/possible clones. Replication-competent clones/possible clones (shown in red) are intact and outgrowth was observed in VOA. Defective clones (shown in purple) were hypermutated and contained stop codons. Non-induced possible clones (shown in blue) did not contain obvious defects but outgrowth of the matching virus was not observed by VOA. About 50–1200 cells within each clone/possible clone were assayed for ca-HIV RNA.

We hypothesized that a large fraction of cells within the clonal populations would have little or no viral RNA, possibly allowing them to survive and proliferate despite infection. To understand the persistence of the clonal populations of infected cells on ART and, in the case of AMBI-1, their contribution to low level viremia during ART, we performed CARD-SGS (Wiegand et al., 2017) and used the data to estimate the fraction of PBMC with detectable levels of viral RNA in the three different categories of possible clones. We also used CARD–SGS to determine the levels of ca-HIV RNA in single PBMC and in single naïve (or stem cell-like memory cells), CD27+ memory [central or transitional memory (CTM)], and CD27- memory [effector memory (EM)] cells for each of the clonal populations. For each clonal population, the fraction of infected PBMC with ca-HIV RNA and the levels of ca-HIV RNA were calculated as described in the CARD-SGS methods paper by Wiegand et al. (2017) and the results are shown in Figure 4, Table 1, and Supplementary Table S1.

**TABLE 1:** Fraction of proviruses expressing ca-HIV RNA and levels of expression in single cells.

**Population type**	**Median fraction of cells expressing HIV RNA (range)**	**Mean number of ca-HIV RNA copies in single cells (range)**	**Fraction of high-expressing cells (# of cells with >20 ca-HIV RNA molecules/# cells with any # of ca-HIV RNA)**
Clones with intact proviruses (*N* = 3)^a^	2.3% (1.2–8.8%)	2.3 (1–10)	0/35
Clones with defective proviruses (*N* = 5)	3.5% (0.9–7.0%)	1.3 (1–4)	0/22
Non-induced clones (*N* = 26)	6.6% (1.3–64.9%)	2.0 (1–16)	0/277
Cells with ca-HIV RNA detected but no DNA detected^b^	>2.2%	1.5 (1–65)	9/784^c^
Cells with DNA detected but no ca-HIV RNA detected	<1.7%	0	0/72

**^*a*^Excluding DR-OG, which may reflect spreading infection instead of clonal amplification. ^*b*^Including both wildtype and drug resistant populations. ^*c*^All 9 high expressing cells (where no DNA was detected) were found in the drug resistant population.**

Although the methods are described in detail and the protocol provided in the CARD-SGS methods paper (Wiegand et al., 2017), we briefly describe the methods here as well. First, like other SGS assays, limiting-dilution PCR is used to determine the endpoint. In this case, it is used to determine the endpoint for infected cells with *gag-pol* DNA and for infected cells with ca-*gag-pol* RNA (referred to as ca-HIV RNA in this manuscript). The cells are then divided into aliquots at a near endpoint for infected cells with ca-HIV RNA (73 aliquots in this case to assay >10,000 infected cells). Because Patient 1 had a very high HIV diversity (2.9% in P6-PR-RT) making it unlikely to obtain two identical ca-HIV RNA sequences that are not from a single cell when only 10 cells with ca-HIV RNA are assayed, we were able to use a near endpoint of ∼10 infected cells with ca-HIV RNA in each aliquot. However, when the HIV diversity of a sample is closer to 1%, then ∼5 cells with ca-HIV RNA is more appropriate and when the diversity is closer to 0.5%, then a true endpoint is used (<1 infected cell with ca-HIV RNA per aliquot). After the aliquots are generated, genomic DNA is extracted from one aliquot and proviral SGS is performed (in this case on P6-PR-RT). Ca-HIV RNA is extracted from the remaining aliquots (72 in this case). RNA from each aliquot is then converted to cDNA and the entire cDNA contents from each aliquot is spread across 96-well plates so that <<1 HIV cDNA molecule is in a single well. The contents of 96-well plates are then PCR amplified and Sanger sequenced as is typical for SGS. After sequencing, identical RNA variants within an aliquot of cells are assumed to have derived from a single cell. For example, if 10 different RNA variants are detected in a single aliquot, then we conclude that 10 cells in that aliquot contained ca-HIV RNA. If 5 copies of one of those variants is detected, then we conclude that one of the 10 single cells with ca-HIV RNA contained 5 copies of RNA. Our previous study demonstrated that CARD-SGS is capable of detecting a single RNA molecule in a single infected cell and a diagram of the assay workflow is shown (Wiegand et al., 2017).

After performing the CARD-SGS assay as described above, we found that intact clones had a median fraction of 2.3% (range 1–9%) of infected PBMC with detectable levels of ca-HIV RNA and a mean level of 2 (range 1–10) ca-HIV RNA molecules per RNA-containing cell (Figure 4, red bars). Defective clones had a median fraction of 3.5% (1–7%) with ca-HIV RNA and a mean level of 1 (range 1–4) ca-HIV RNA molecule per RNA-containing cell (Figure 4, purple bars). The clones that were not induced in VOA (non-induced possible clones) had a median fraction of 6.6% (1–65%) and a mean level of 2 (range 1–16) ca-HIV RNA molecules per RNA-containing cell (Figure 4, blue bars). Thus, no significant difference in the fractions or levels of P6-PR-RT ca-HIV RNA was observed between intact, defective, and the non-induced clones (*p* = 0.20–0.99 Mann Whitney). This result is consistent with a study by Pollack et al. (2017) demonstrating that cells containing defective proviruses can express HIV transcripts, resulting in the recognition and clearance by CD8+ T cells *ex vivo*. Ca-HIV RNA matching 13 of the 34 cell clones was detected in 250,000–350,000 sorted CTM and EM cells (indicated in Figure 4). All ca-HIV RNA detected in naïve cells harbored drug resistance mutations and, hence, did not match any of the WT cell clones. As discussed above, the drug-resistance mutations in this donor appear to have arisen on a CXCR4 backbone, explaining their higher infectivity of naïve cells that have higher CXCR4 surface expression compared to CTM and EM (Nicholson et al., 2001). In total, the fraction of naïve, CTM, and EM cells with ca-HIV RNA was not different from the total PBMC (4% of infected naïve cells with ca-HIV RNA, 2% of CTM, and 9% of EM). However, infected EM cells had a significantly higher fraction with ca-HIV RNA than infected CTM cells (7.8 × 10^–10^ Chi-sq). Consistent with the findings of others (Grau-Exposito et al., 2017), higher levels of ca-HIV RNA were also observed in some single EM cells but the difference did not achieve significance with our level of sampling (*p* = 0.1 Kruskal-Wallis).

Despite the AMBI-1 clone’s being the source of WT persistent viremia in this individual (Simonetti et al., 2016), only a small fraction of cells (2.3%) within the AMBI-1 clone contained ca-HIV RNA, strongly suggesting that clonal populations persist because the vast majority of the daughter cells at each division produce little or no HIV RNA. These data demonstrate that all the cells within a clone are not uniformly producing HIV RNA. These results also suggest that the production of infectious virus by a small fraction of the cells in the clonal populations that carry intact proviruses is likely to be the source of rapid rebound viremia when ART is interrupted. Likewise, only a small fraction of the clonal cell siblings appear to be the source of viral outgrowth in the VOA: about 3.6% of the AMBI-1 infected CD4+ T cells produced infectious virus in the quantitative VOA (Simonetti et al., 2016). Since it is improbable that the small numbers of unspliced viral RNA copies detected per cell are sufficient to support infectious virus production, it is likely that a much smaller fraction of cells with intact proviruses are actually producing virions at any one time *in vivo*. Taken together, these data support that only a small fraction of proviruses in possible proviral clones are actively being transcribed, independent of intactness or outgrowth potential. Conversely, about 90% or more of the infected cells within clonally expanded populations contain little or no detectable unspliced viral RNA at a given point in time, allowing these cells to proliferate and survive for years, even though they may contain fully intact, infectious proviruses. These data further suggest, although more studies are needed to test the hypothesis, that T cells are able to be activated to proliferate without inducing the expression of the integrated provirus.

### Sources of Drug Resistant Plasma Virus During ART

**FIGURE 5:**
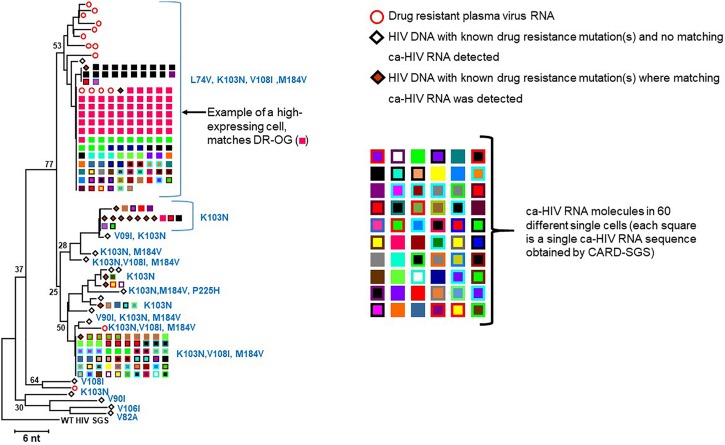
NJ distance tree of drug-resistant variants. Plasma SGS are shown as red, open circles, PBMC proviruses with matching ca-HIV RNA are shown as orange diamonds, ca-HIV RNA in single cells are shown in different colored squares, PBMC proviruses with no ca-HIV RNA detected are open diamonds. The number of single cells that contained ca-HIV RNA is indicated by the number of different colored squares. The number of ca-HIV RNA molecules in each single cell is indicated by the number of squares of each color. One cell that contained a high level of ca-HIV RNA was detected (bright pink squares) and the sequence of this ca-HIV RNA matched the sequence of the most prevalent variant in the plasma and the sequence of the Drug Resistant Outgrowth-1 variant. Only RNA sequences identical to DR proviral DNA sequences are shown.

To compare the DR variants in the proviral population to those in the plasma, we constructed a NJ tree (Figure 5) of all DR proviral sequences (diamonds) and DR plasma variants (circles) and identified the mutations in each variant (defined by the Stanford University HIV Drug Resistance Database)^2^. The DR ca-HIV RNA sequences with matching HIV DNA are also shown in Figure 5 (squares) (discussed later). As in Figure 2, all the DR viral DNA sequences formed a distinct node relative to the WT sequences, implying a common evolutionary origin, which likely arose during one of the treatment interruptions (Figure 1A). Almost all the DR plasma sequences contained four linked mutations in RT (L74V, K103N, V108I, M184V) which, together, conferred resistance to each drug in the contemporaneous ART regimen. However, of 30 proviral DR variants, only three had all four of these linked DR mutations (Figure 5). This observation suggests that, perhaps, as many as 90% of the DR proviral sequences are not making a significant contribution to the plasma viral RNA pool. By contrast to the viral DNA, 13 of the 15 plasma DR viral RNA sequences belonged to the node with all four linked resistance mutations. Of the two sequences that did not have all four mutations, one had K103N alone and the other had K103N linked to V108I and M184V. Overall, the presence of the four linked DR mutations in only three out of a total of 183 proviruses implies that, at the time the sample was taken, less than 2% of the infected cells were the likely source of the replicating DR plasma virus.

The fraction of cells with detectable ca-HIV RNA in the WT versus DR populations is compared in Table 2. At the timepoint analyzed, an average of 4.7% of cells infected with WT proviruses had ca-HIV RNA while 17.2% of cells with DR proviruses had ca-HIV RNA (*p* = 3 × 10^–14^). Of the cells that carried proviruses that had the four DR mutations, 11.0% had ca-HIV RNA (detected by either DNA with matching ca-RNA as shown in Figure 4 or detected as only ca-HIV RNA in single cells (not shown in Figure 4 but included in sequences submitted to genbank accession numbers MK148701-MK152485). These results are consistent with our hypothesis that, despite ongoing viral replication of at least some of the DR variants, the majority of cells that carried the DR proviruses contained little or no viral RNA and, again, that only a small fraction of infected cells contribute to plasma viremia at a given time. Figure 5 shows the DR ca-HIV RNA variants for which we detected the matching proviruses among the 30 that were sequenced. Eleven percent of the DR proviruses with ca-HIV RNA had all four of the DR mutations discussed previously, suggesting that some of these proviruses may be in newly infected cells and may be contributing to the DR plasma virus.

**TABLE 2:** Mean fraction of infected cells containing ca-HIV RNA and levels of ca-HIV RNA in single cells: wildtype versus drug resistant populations.

**Population type (# of cells assayed)**	**Mean fraction of expressing proviruses**	***p*-value^b^**	**Mean number of ca-HIV RNA copies in single cells (range)**	***p*-value^b^**	**High-expressing cells (# of cells with > 20 ca-HIV RNA molecules/# cells with any # of ca-HIV RNA)**	***p*-value^c^**
Cells with WT proviruses (*N* = 820)	4.7%	3 × 10^–14^	1.6 (1–16)	2 × 10^–4^	0/820	4 × 10^–5^
Cells with DR proviruses (*N* = 324)	17.2%		2.2 (1–65)		9/324	
Cells with all 4 DR mutations (*N* = 116)^a^	11.0%		2.6 (1–65)		5/116	

**^*a*^Subset of all DR proviruses that have linked L74V, K103N, V108I, M184V. ^*b*^Mann-Whitney test.*^*c*^*2-tail Fisher Exact test.**

### Comparison of High Expressing Cells Within Wild-Type and Drug Resistant Populations

**FIGURE 6:**
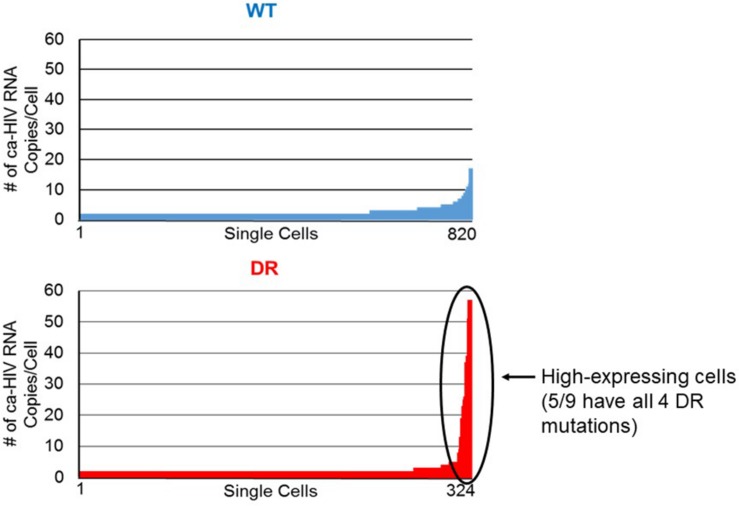
Levels of ca-HIV RNA in single cells that carried the wild-type (blue) and drug resistant (red) proviruses. The number of ca-HIV RNA copies per cell in the wild-type population ranged from 1 to 16. The number of ca-HIV RNA copies per cell in the drug resistant population ranged from 1 to 65. Data were analyzed from 820 cells with wild-type proviruses and 324 cells with drug resistant proviruses. The remaining infected cells did not contain any detectable ca-HIV RNA. The high-expressing cells are indicated with the black arrow.

We also compared the distribution of ca-HIV RNA levels in single infected cells for the WT (*N* = 820) and DR (*N* = 324) variants (Table 2, Supplementary Table S2, and Figure 6). The cells with WT variants had levels of ca-HIV RNA ranging from 1 to 16 copies/single PBMC with ca-HIV RNA (mean = 1.6), whereas the DR population had levels ranging from 1 to 65 copies/single PBMC with ca-HIV RNA (mean = 2.2) (*p* = 2 × 10^–4^) (Table 2). Cells with greater than 20 ca-HIV RNA copies, were observed more frequently in the DR variants (0 cells in WT, 9 cells in DR, Tables 1, 2 and Figure 6). One example of a cell with high levels of ca-RNA is shown in the NJ tree in Figure 5 (pink squares, indicated with a black arrow). Five of the 9 cells with high levels of viral RNA contained proviruses with all four linked DR mutations. It is likely that some or all of these cells are producing virus and may, therefore, be short-lived and, hence, rarely detected. The remaining 4 high-expressing proviruses contained the following DR mutations: (1) K103N, V108I, M184V, H221Y, (2) K103N, V108I, M184V, V90I, (3) K103N, V108I, M184V, V90I, M46I, and (4) K103N, M184V, D67N. This hypothesis is consistent with a higher frequency of high expressing cells detected in the multi-DR population versus WT.

## Conclusion

To develop effective HIV-1 cure strategies, it is critically important to define how HIV-1 infected cells persist and proliferate in patients on ART, especially those cells that contain intact proviruses and can be the source of persistent, low-level viremia during ART and cause viral rebound when ART is interrupted. We present detailed evidence in one very well-characterized individual that most (>90%) of individual cells within infected cell clones carrying replication-competent proviruses are transcriptionally silent, independent of the T-cell subset, possibly allowing them to escape targeting by the immune system and direct cytopathic effects caused by the virus. The life-span and clearance rate of the small fraction of cells with intact proviruses that express ca-HIV RNA is not defined but we hypothesize that they are eliminated and may be replaced by reactivation of siblings in the same clone. Testing of this hypothesis will require new approaches to assess single cell turnover *in vivo* of specific cell clones that constitute a very small fraction of all clonal CD4+ T cell populations. The observation that, in general, a small fraction of cells within infected T cell clones contain unspliced HIV RNA, including clones with intact, infectious proviruses, suggests that T cells can be activated to proliferate without inducing the expression of HIV proviruses. The limitations of this observation, however, must be noted. Although 34 different possible cell clones were analyzed, all were within the same individual. Variation across individuals may exist. Furthermore, possible cell clones were identified by the detection of identical sub-genomic sequences and not by their sites of integration into the host genome. It is known that identical sequences can result from bottlenecks imposed at transmission or for the selection of specific mutations, such as those that confer immune escape. Therefore, future studies using new methods, like those described by Einkauf et al. (2019), are needed to assess the “true” clonality of proviruses with identical subgenomic sequences.

The DR virus, which comprised about half of the virus present in the low-level viremia in the donor at the timepoint we sampled, consisted of cells with relatively high levels of ca-HIV RNA, defined here as having greater than 20 individual unspliced ca-HIV RNA molecules per cell. No such cells were found in the infected cells that carried WT proviruses, consistent with a previous study that suggested that cells with high levels of viral RNA may be more common in untreated individuals than in those on ART (Wiegand et al., 2017), as expected. We found one match between plasma RNA sequences and a cell with high levels of ca-HIV RNA, suggesting that high-expressing cells may be a source of plasma virus. This finding supports the idea that cells with high levels of viral RNA may be associated with viral replication.

Overall, the results of this study provide direct evidence that HIV-1 infected cell clones are able to persist despite ART because a high fraction of the cells in these clones have transcriptionally-silent proviruses. The results also show that latently-infected cells within clones harboring intact proviruses exist in multiple CD4+ memory subsets and are an important component of the HIV-1 reservoir, however, only a small fraction of each clone is transcriptionally-active at any one time, and potentially capable of producing infectious virus that can seed rebound if ART is stopped. In depth characterization of the HIV-1 reservoir and latently-infected cell clones, as performed here, may help define the appropriate goals and therapeutic approaches to achieve an HIV-1 cure in the future.

## Materials and Methods

### Donor and Samples in Study

One hundred million PBMC were obtained from the timepoint indicated in red in Figure 1 from “Patient 1” in Maldarelli et al. (2014) and Simonetti et al. (2016). Plasma sequences and a subset of the proviral sequences from the same timepoint and the results of the VOA are described in Simonetti et al. (2016).

### Cell-Associated RNA and DNA-Single Genome Sequencing (CARD-SGS)

CARD-SGS was performed on P6-PR-RT exactly as described in Wiegand et al. (2017) on a total of 73 aliquots of infected cells. In brief, CARD-SGS is single-genome sequencing (SGS) of the unspliced ca-HIV RNA in aliquots of cells at a near endpoint for those with unspliced ca-HIV RNA. For example, aliquots containing ∼200 infected cells may be used, each of which may contain only ∼10 cells with ca-HIV RNA. RNA originating from different cells can be distiguished by their unique sequences (after SGS) when a sample with high HIV diversity is used. In the example above, ∼10 different HIV RNA variants would be identified by SGS. The number of sequences detected from each of the 10 variants reflects the level of RNA expression in the different single cells. Using single ACH2 cells, we previously showed that our method can detect a single HIV-1 P6-PR-RT RNA molecule in a single cell (Wiegand et al., 2017).

The number of infected cells in each aliquot was determined by HIV DNA copy number measured by the iCAD assay (Hilldorfer et al., 2012) and the frequency of cells from each of the 34 different possible clones in each aliquot was determined by their DNA sequences after SGS. For example, the AMBI-1 clone was found to comprise 11% of the total proviral population by SGS and the number of total infected cells in each aliquot by iCAD was about 200. Therefore, each aliquot contained ∼20 AMBI-1 infected cells, of which, <1 per aliquot was found to have ca-HIV RNA after performing CARD-SGS. Therefore, the number of aliquots that were positive for AMBI-1 ca-HIV RNA after performing CARD-SGS could be used to calculate the fraction of AMBI-1 infected cells that have ca-HIV RNA.

Because SGS on HIV-1 RNA is known to include errors introduced in the RT-PCR step (Palmer et al., 2005), variants that differed by a single nucleotide from a group of identical sequences of 5 or more that did not appear more than once were not considered to be a different variant, as previously described (Wiegand et al., 2017). Replication-competent proviruses were defined as P6-PR-RT sequences that were identical matches to variants that grew out in the VOA. Defective proviruses were those that contained stop codons or other obvious defects. Proviruses termed “non-induced” did not contain any obvious defects (i.e., stop codons) in the P6-PR-RT region analyzed but no outgrowth was observed in VOA at our level of sampling. A total of about 10,450 HIV infected cells were analyzed in the 72 aliquots. Sequences obtained by CARD-SGS were aligned using ClustalW^3^. MEGA6^4^ was used to construct NJ trees to match ca-HIV RNA sequences to the proviral DNA variants. Drug resistant variants were identified using the Stanford Database hivdb.stanford.edu). Sequences obtained by CARD-SGS are available in genbank^5^ accession #’s MK148701-MK152485.

### Viral Outgrowth Assays

Quantitative viral outgrowth assays (QVOA) was performed, as described previously (Siliciano and Siliciano, 2005), with the following modifications: CD4+ cells were isolated from Cryopreserved PBMC by negative selection (Stemcell Technologies), serially diluted threefold from 1,000,000 cells per well to 30,000 cells per well, and seeded in individual wells in 6 replicates. Cells were stimulated with PHA overnight and a 10-fold excess of allogeneic irradiated feeder cells obtained from uninfected donors was added. CD4+ lymphoblasts from HIV-1-negative donors were added, and virus co-cultures were carried out for 3 weeks. Supernatants were tested weekly to measure HIV-1 p24 antigen by ELISA (Perkin Elmer). Aliquots of the supernatants were also frozen for sequencing the recovered viruses.

### Sorting CD4+ T Cell Subsets

Peripheral blood cells were obtained by leukapheresis. Following lysis of red blood cells, they were preserved in freezing medium at -80C/LN. 3.0 × 10^8^ PBMC (93.1% viability) were thawed and stained with LIVE/DEAD Aqua stain (Molecular Probes, L34957, 5 min, 22°C), CD3-APC-H7 (BD Biosciences, 641406), CD4-BV785 (BioLegend, 317442), CD8-QDot655 (Invitrogen, Q10055), CD11c-PE (BD Biosciences, 347637), CD14-PE (BD Biosciences, 555398), CD27-PE/Cy5 (Beckman Coulter, 6607107), CD45RO-ECD (Beckman Coulter, IM2712U), CD56-APC (BioLegend, 304610), CD57-BV421 (VRC Ab), CCR7-Ax700 (VRC Ab), and TCR γδ (BD Biosciences, 555718) for 15 min at 22°C. Cells were washed, kept on ice, and sorted on a BD FACSAria into CD4 memory subsets as follows: Naïve (CD3+ Aqua-CD8-CD4hiCD56-TCRγδ-CD14-CD11c-CD27 + CD45RO-CCR7 + CD57-), Central/Transitional Memory (CTM, CD3+ Aqua-CD8-CD4hiCD56-TCRγδ-CD14-CD11c-CD27+ CD45RO+), and Effector Memory (EM, CD3 + Aqua-CD8-CD4hiCD56-TCRγδ-CD14-CD11c-CD27-) (Supplementary Figure S1).

### Statistics

Standard statistical analyses were performed with the use of packages in R. The statistical tests used are indicated in the text and/or the tables.

## Data Availability Statement

The datasets generated and analyzed during the current study are available in GenBank accession numbers MK148701-MK152485.

## Ethics Statement

The donor was enrolled in NIH protocol 00-I-0110 conducted at the NIH Clinical Center in Bethesda, MD. The study was approved by the NIH Internal Review Board. The sample was collected specifically for this study and the donor provided written informed consent to participate in this study.

## Author Contributions

AM conducted the experiments and wrote the manuscript. JS, MS, AW, CR, BK, FS, EB, LP, and DC-V conducted the experiments. MB, WS, and AC analyzed the data. SH, JM, and JC conceived the idea and wrote the manuscript. MK conceived the idea, analyzed the data, and wrote the manuscript. FM was the PI on the clinical protocol and conceived the idea. SP wrote the manuscript.

## Conflict of Interest

JM was a consultant to Gilead Sciences and a shareholder of Co-crystal, Inc. The remaining authors declare that the research was conducted in the absence of any commercial or financial relationships that could be construed as a potential conflict of interest.

## Footnotes

1 https://michaelbale.shinyapps.io/prob_identical

2 https://hivdb.stanford.edu/

3 http://www.ebi.ac.uk/Tools/msa/clustalw2/

4 www.megasoftware.net

5 www.ncbi.nlm.nih.gov/genbank

## Supplementary Material

FIGURE S1 Gating for CD4 T Naïve, CTM, and EM Cell Subsets from PBMC **(A)** not part of multi-cell conjugates **(B)** that were viable and stained with the T cell markers CD3 **(C)** and CD4 **(D)** but not myeloid cell markers CD14 and CD11c, or lineage markers CD56 and TCR γδ **(E)** were divided by CD27 and CD45RO staining and collected as T_*CTM*_ (**F**, top-right gate) or T_*EM*_ (**F**, bottom gate) subsets. Cells that were single-positive (T_27__+_; **F**, top-left gate) for CD27 were further divided by CD57 and CCR7 staining to collect T_*N*_ **(G)** subset. Numbers on plots represent percentages of plotted cells falling within the gates shown. Naïve CD4 defined as CD45RO-, CD27+, CCR7+, CD57-. CTM CD4 defined as CD45RO+, CD27+. EM CD4 defined as CD45RO±, CD27-.

FIGURE S2 NJ distance tree of drug-resistant sequences from longitudinal samples from Patient 1. The tree show that drug resistance mutations emerged years prior to virologic failure. ^∗^Bootstrap values >75.

TABLE S1 Fraction of cells in clones and probable clones that contain unspliced ca-HIV RNA.

TABLE S2 Levels of unspliced ca-HIV RNA in cells with proviruses containing major drug resistance mutations.
