# Supplementary figures and images for: HIV Infected T Cells Can Proliferate in vivo Without Inducing Expression of the Integrated Provirus
Source: Front Microbiol. 2019 Oct 1;10:2204. doi: 10.3389/fmicb.2019.02204 (PMC6781911; doi:10.3389/fmicb.2019.02204)

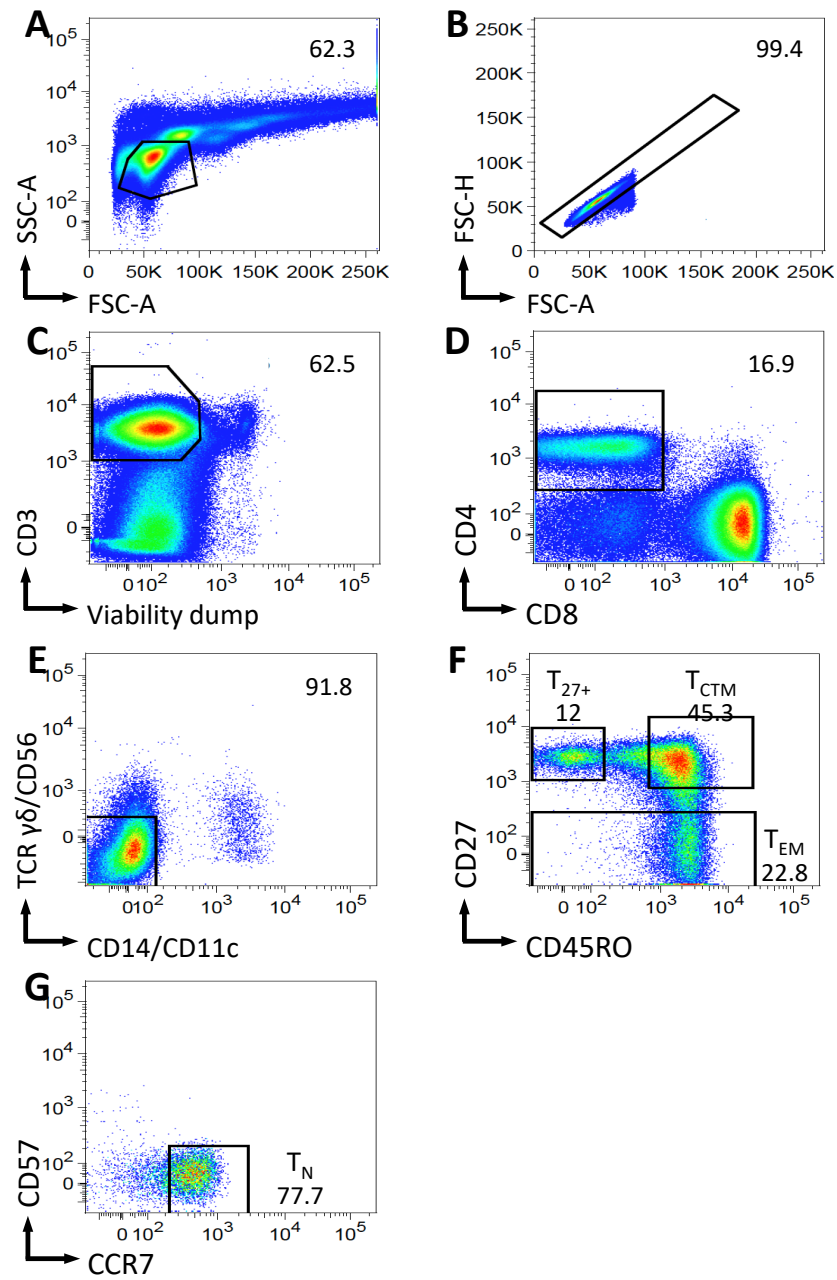

Supplemental Figure 1

Supplement: FIGURE S1 — Gating for CD4 T Naïve, CTM, and EM Cell Subsets from PBMC (A) not part of multi-cell conjugates (B) that were viable and stained with the T cell markers CD3 (C) and CD4 (D) but not myeloid cell markers CD14 and CD11c, or lineage markers CD56 and TCR γδ (E) were divided by CD27 and CD45RO staining and collected as TCTM (F, top-right gate) or TEM (F, bottom gate) subsets. Cells that were single-positive (T27+; F, top-left gate) for CD27 were further divided by CD57 and CCR7 staining to collect TN (G) subset. Numbers on plots represent percentages of plotted cells falling within the gates shown. Naïve CD4 defined as CD45RO-, CD27+, CCR7+, CD57-. CTM CD4 defined as CD45RO+, CD27+. EM CD4 defined as CD45RO±, CD27-. [file Data_Sheet_1.PDF]

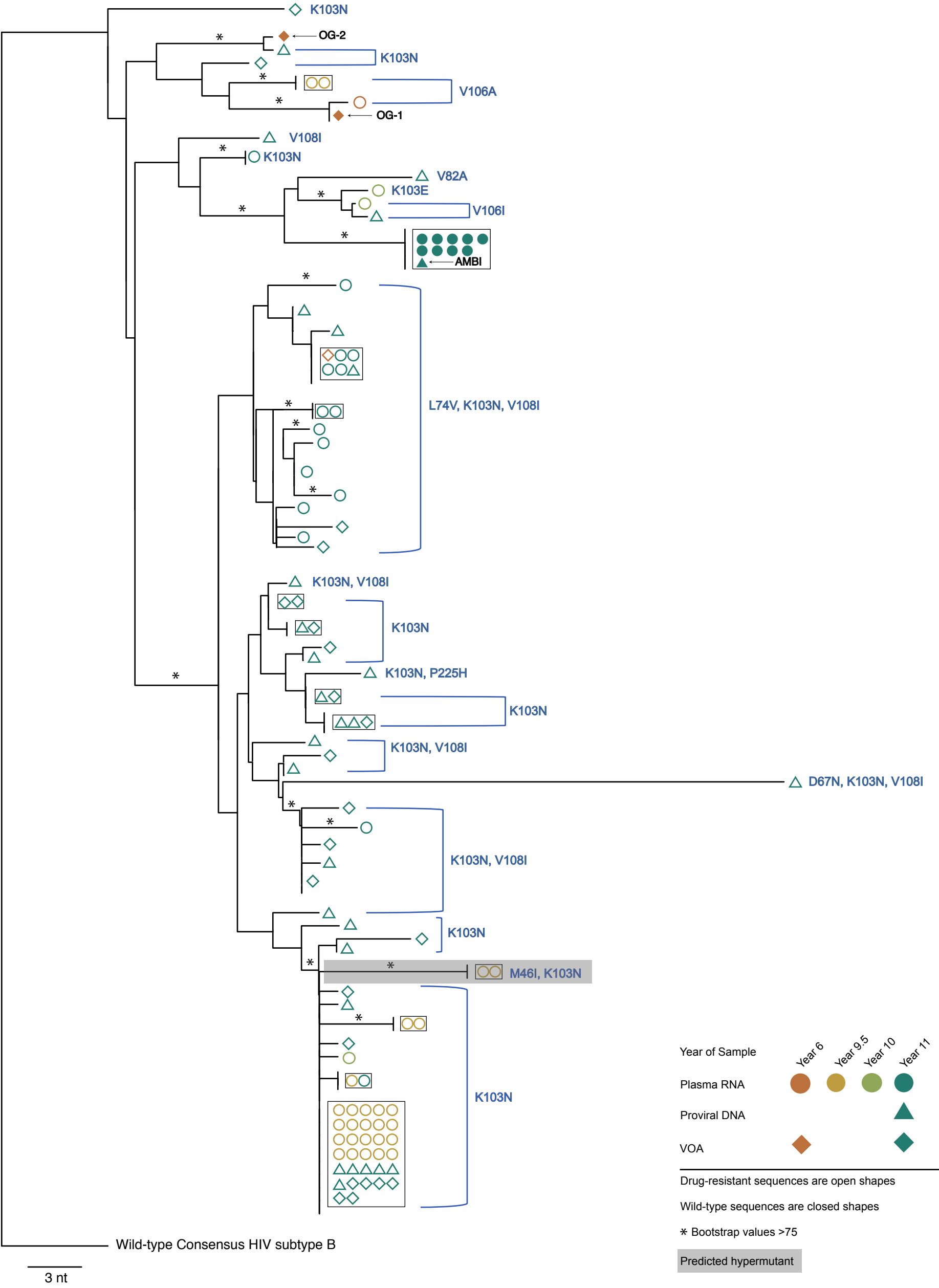

Supplemental Figure 2

Supplement: FIGURE S2 — NJ distance tree of drug-resistant sequences from longitudinal samples from Patient 1. The tree show that drug resistance mutations emerged years prior to virologic failure. ∗Bootstrap values >75. [file Data_Sheet_2.PDF]
