# Supplementary material for: HIV Infected T Cells Can Proliferate in vivo Without Inducing Expression of the Integrated Provirus
Source: Front Microbiol. 2019 Oct 1;10:2204. doi: 10.3389/fmicb.2019.02204 (PMC6781911; doi:10.3389/fmicb.2019.02204)
Supplement: TABLE S1 — Fraction of cells in clones and probable clones that contain unspliced ca-HIV RNA. [file Table_1.DOCX]

Table S1. Fraction of cells in clones and probable clones that contain unspliced ca-HIV RNA

| Clone Type | Clone ID | Number of infected cells with HIV RNA/total cells analyzed for each clone (%) | Average number of RNA molecules per expressing cell | Number of cells with the # of RNA molecules indicated  (# of RNA molecules) |
| --- | --- | --- | --- | --- |
| Intact Clones | 1 (AMBI-1) | 28/1199 (2.3%) | 2.5 | 12(1), 5(2), 6(3), 2(4), 1(5), 1(6), 1(10) |
|  | 2 (WT Outgrowth-1) | 2/171 (1.2%) | 1 | 2(1) |
|  | 3 (WT Outgrowth-2) | 5/57 (8.8%) | 1.6 | 3(1), 1(2), 1(3) |
| Defective Clones | 4 | 1/114 (0.9%) | 1 | 1(1) |
|  | 5 | 4/288 (1.8%) | 1.3 | 3(1), 1(2) |
|  | 6 | 2/57 (3.5%) | 1 | 2(1) |
|  | 7 | 11/171 (6.4%) | 1.5 | 8(1), 1(2), 1(3), 1(4) |
|  | 8 | 4/57 (7.0%) | 1.3 | 3(1), 1(2) |
| Non-Induced Clones | 9 | 3/228 (1.3%) | 1 | 3(1) |
|  | 10 | 1/57 (1.8%) | 1 | 1(1) |
|  | 11 | 1/57 (1.8%) | 1 | 1(1) |
|  | 12 | 2/114 (1.8%) | 6 | 1(1), 1(11) |
|  | 13 | 1/57 (1.8%) | 1 | 1(1) |
|  | 14 | 1/57 (1.8%) | 1 | 1(1) |
|  | 15 | 1/57 (1.8%) | 1 | 1(1) |
|  | 16 | 10/343 (2.9%) | 1 | 10(1) |
|  | 17 | 5/171 (2.9%) | 1 | 5(1) |
|  | 18 | 2/57 (3.5%) | 1 | 2(1) |
|  | 19 | 2/57 (3.5%) | 1 | 2(1) |
|  | 20 | 2/57 (3.5%) | 1 | 2(1) |
|  | 21 | 7/114 (6.1%) | 1.2 | 5(1), 2(2) |
|  | 22 | 4/57 (7.0%) | 1 | 4(1) |
|  | 23 | 17/228 (7.5%) | 1.3 | 14(1), 1(2), 2(3) |
|  | 24 | 23/286 (8.0%) | 1.6 | 14(1), 5(2), 3(3), 1(4) |
|  | 25 | 13/114 (11.4%) | 1.5 | 8(1), 3(2), 2(3) |
|  | 26 | 14/114 (12.3%) | 1.6 | 10(1), 1(2), 2(3), 1(4) |
|  | 27 | 19/114 (16.7%) | 1.1 | 18(1), 1(2) |
|  | 28 | 38/228 (16.7%) | 2.8 | 15(1), 11(2), 4(3), 3(4), 1(5), 1(6), 1(7), 1(9), 1(10) |
|  | 29 | 10/57 (17.5%) | 1.3 | 8(1), 1(2), 1(3) |
|  | 30 | 11/57 (19.3%) | 1.5 | 7(1), 3(2), 1(3) |
|  | 31 | 13/57 (22.8%) | 1.2 | 12(1), 1(3) |
|  | 32 | 19/57 (33.3%) | 2.2 | 12(1), 3(2), 2(3), 1(8), 1(9) |
|  | 33 | 20/57 (35.1%) | 1.9 | 12(1), 4(2), 1(3), 2(4), 1(6) |
|  | 34 | 37/57 (64.9%) | 3.9 | 11(1), 3(2), 4(3), 7(4), 6(5), 1(6), 1(7), 1(8), 1(9), 1(11), 1(16) |

Table S2. Levels of unspliced ca-HIV RNA in cells with proviruses containing major drug resistance mutations

| Drug Resistance Variant ID | Average number of RNA molecules per cell | Number of cells (# of RNA molecules) |
| --- | --- | --- |
| 1 (Drug Resistant Outgrowth) | 3.2 | 25(1), 7(2), 2(3), 2(4), 1(12), **1(56)^a^** |
| 2 | 1.7 | 22(1), 6(2), 3(3), 4(4) |
| 3 | 5.3 | 3(1), **1(24)** |
| 4 | 1 | 5(1) |
| 5 | 1 | 4(1) |
| 6 | 1 | 4(1) |
| 7 | 1 | 2(1) |
| 8 | 1 | 1(1) |

**^a^ Bold** indicates a high-expressing cell (>20 ca-HIV RNA molecules per cell).
